# A charge-transfer salt, 3,5-dimethyl-1-(4-nitro­benz­yl)pyridinium 7,7,8,8-tetra­cyano­quinodimethane

**DOI:** 10.1107/S1600536808008258

**Published:** 2008-04-04

**Authors:** Min Wang, Hong-Bo Zhou, You-Cun Chen

**Affiliations:** a Anhui Key Laboratory of Functional Coordination Compounds, School of Chemistry and Chemical Engineering, Anqing Normal University, Anqing 246003, People’s Republic of China

## Abstract

In the title salt, C_14_H_15_N_2_O_2_^+^·C_12_H_4_N_4_^−^, the asymmetric unit contains one cation and one anion. C—H⋯N and C—H⋯N and C—H⋯O hydrogen bonds and π–π stacking inter­actions (inter­planar distance 3.845 Å) are found in the crystal structure.

## Related literature

For general background, see: Madalan *et al.* (2002 ▶); Ren, Chen *et al.* (2002 ▶); Ren *et al.* (2003 ▶); Ren, Meng *et al.* (2002 ▶). For related literature, see: Liu *et al.* (2005 ▶); Wang *et al.* (2006 ▶).
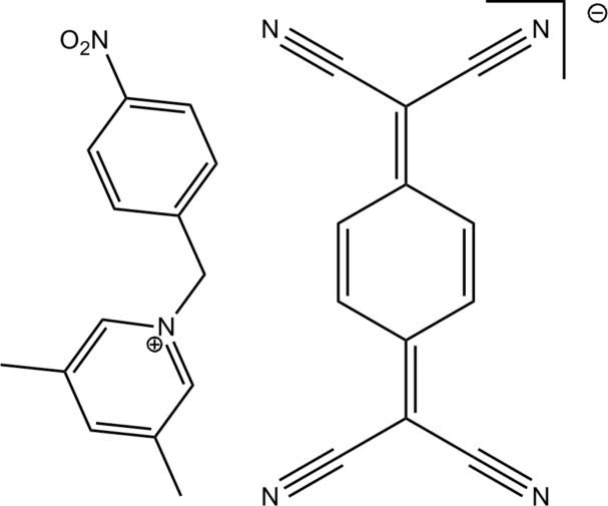


## Experimental

### 

#### Crystal data

C_14_H_15_N_2_O_2_^+^·C_12_H_4_N_4_^−^
*M* *_r_* = 447.47
Triclinic, 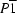

*a* = 8.098 (2) Å
*b* = 9.137 (2) Å
*c* = 16.542 (4) Å
α = 76.194 (3)°
β = 75.951 (3)°
γ = 86.933 (3)°
*V* = 1153.0 (5) Å^3^
*Z* = 2
Mo *K*α radiation
μ = 0.09 mm^−1^
*T* = 293 (2) K
0.18 × 0.12 × 0.10 mm

#### Data collection

Bruker SMART APEX CCD diffractometer
Absorption correction: multi-scan (*SADABS*; Bruker, 2000 ▶) *T*_min_ = 0.985, *T*_max_ = 0.992
5765 measured reflections
3998 independent reflections
3255 reflections with *I* > 2σ(*I*)
*R*_int_ = 0.018

#### Refinement

*R*[*F* ^2^ > 2σ(*F* ^2^)] = 0.046
*wR*(*F* ^2^) = 0.144
*S* = 1.00
3998 reflections
309 parameters
H-atom parameters constrained
Δρ_max_ = 0.15 e Å^−3^
Δρ_min_ = −0.20 e Å^−3^

### 

Data collection: *SMART* (Bruker, 2000 ▶); cell refinement: *SAINT-Plus* (Bruker, 2000 ▶); data reduction: *SAINT-Plus*; program(s) used to solve structure: *SHELXS97* (Sheldrick, 2008 ▶); program(s) used to refine structure: *SHELXL97* (Sheldrick, 2008 ▶); molecular graphics: *SHELXTL* (Sheldrick, 2008 ▶); software used to prepare material for publication: *SHELXTL*.

## Supplementary Material

Crystal structure: contains datablocks I, global. DOI: 10.1107/S1600536808008258/cf2189sup1.cif

Structure factors: contains datablocks I. DOI: 10.1107/S1600536808008258/cf2189Isup2.hkl

Additional supplementary materials:  crystallographic information; 3D view; checkCIF report

## Figures and Tables

**Table 1:** Hydrogen-bond geometry (Å, °)

*D*—H⋯*A*	*D*—H	H⋯*A*	*D*⋯*A*	*D*—H⋯*A*
C5—H5⋯N2	0.93	2.56	2.895 (2)	102
C7—H7*B*⋯N4^i^	0.97	2.43	3.245 (3)	141
C8—H8⋯O2^ii^	0.93	2.46	3.119 (2)	128

Symmetry codes: (i) 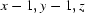; (ii) 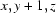.

**Fig. 1.**
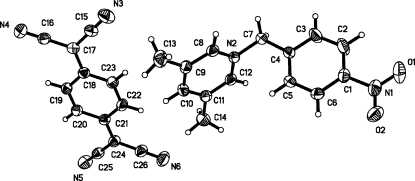
The asymmetric unit, with the atom-numbering scheme. Displacement ellipsoids are drawn at the 30% probability level.

**Fig. 2.**
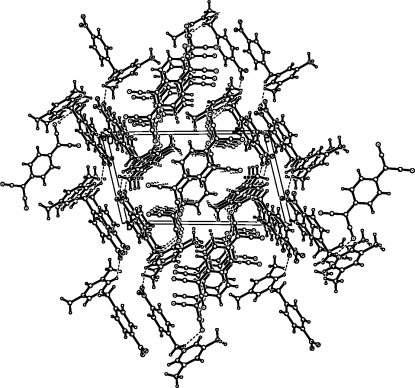
A packing diagram for (I). Hydrogen bonds are shown as dashed lines.

**Fig. 3.**
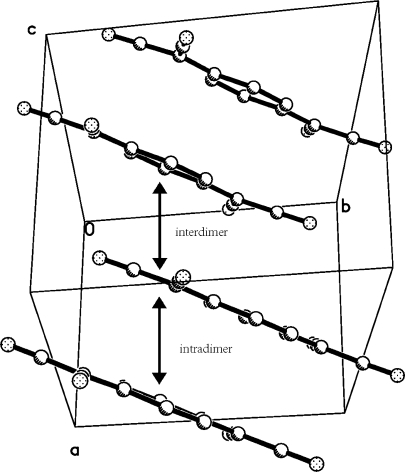
A side-view of the one-dimensional anionic stack of (I).

## supplementary crystallographic information

### Comment

Recently, using benzylpyridinium derivatives ([RBzPy]^+^ where R represents a substituent group) with flexible molecular configuration as a counter-cation to control the arrangement of anions [M(mnt)_2_]^-^ (M = Ni, Pd, Pt), a series of ion-pair compounds that show segregated columnar stacks of cations and anions has been prepared (Madalan *et al.*,  2002; Ren, Chen *et al.*, 2002; Ren *et al.*,  2003; Ren, Meng *et al.*, 2002). The radical of TCNQ also shows a planar arrangement and extended electronic structures that are similar to the [M(mnt)_2_]^-^ ion, and has been extensively used to build molecular solids with low-dimensional conductivity and magnetic features, in which the electronic transport and magnetically coupled interactions can be achieved through π–π interactions between radicals along the direction of the radical stack column (Liu *et al.*,  2005; Wang *et al.*, 2006). This character of the TCNQ^-^ ion prompted us to extend our research to a series of [RBzPy][TCNQ] compounds in order to gain more insight into the relationship between the intermolecular cooperation interactions and the magnetic properties of the compounds with low-dimensional structural features. In this paper, we report the crystal structure of the title compound.

The asymmetric unit contains one (C_14_H_15_N_2_O_2_)^+^ cation and one [C_8_H_4_(CN)_4_]^-^ anion (Fig. 1). It stacks as completely segregated columns of TCNQ anions/molecules and 3,5-dimethyl-1-(4-nitrobenzyl)pyridinium cations, as illustrated by the projection along the crystallographic a axis in Fig. 2. The cation and anion columns are linked by hydrogen-bonding interactions. Within an anionic column, a strongly bound [(TCNQ)_2_]^2-^ unit is formed, and adjacent units are displaced relative to each other along the direction of the shorter molecular axis of TCNQ. The benzene rings are parallel to each other. In a TCNQ column, the mean interplanar separations within two different overlapping pairs are 5.745 Å inter-dimer and 3.845 Å intra-dimer, respectively, indicating weak π–π stacking interactions. The (C_14_H_15_N_2_O_2_)^+^ cation has a Λ-shaped conformation, and the dihedral angles formed by the C4/C7/N2 plane with the benzene and pyridinium rings are 4.12 (11) and 80.45 (12)°, respectively.

### Experimental

3,5-Dimethyl-1-(4-nitrobenzyl)pyridinium iodide was prepared by the direct combination of 1:1 molar equivalents of 3,5-dimethyl-1-(4-nitrobenzyl)pyridinium chloride and NaI in a warm solution in acetone at 313 K. A white precipitate was formed (NaCl), which was filtered off, and a white microcrystalline product was obtained by evaporating the filtrate. 1:2 Molar equivalents of 3,5-dimethyl-1-(4-nitrobenzyl)pyridinium iodide and TCNQ were mixed directly in a solution in methanol, and the mixture was refluxed for 12 h. The dark-green microcrystalline product which formed was filtered off, washed with MeOH and dried *in vacuo*. Single crystals of (I) suitable for structure analysis were obtained by diffusing diethyl ether into an acetonitrile solution of (I).

### Refinement

H atoms were positioned geometrically, with C—H = 0.93, 0.97 and 0.96 Å for aromatic, methylene and methyl H atoms, respectively, and constrained to ride on their parent atoms, with *U*_iso_(H) = xUeq(C), where *x* = 1.5 for methyl H and *x* = 1.2 for all other H atoms.

### Crystal data

**Table:**

C_14_H_15_N_2_O_2_^+^·C_12_H_4_N_4_^–^	*Z* = 2
*M**_r_* = 447.47	*F*_000_ = 466
Triclinic, *P*1	*D*_x_ = 1.289 Mg m^−^^3^
Hall symbol: -P 1	Mo *K*α radiation λ = 0.71073 Å
*a* = 8.098 (2) Å	Cell parameters from 3033 reflections
*b* = 9.137 (2) Å	θ = 2.3–27.9º
*c* = 16.542 (4) Å	µ = 0.09 mm^−^^1^
α = 76.194 (3)º	*T* = 293 (2) K
β = 75.951 (3)º	Pillar, purple
γ = 86.933 (3)º	0.18 × 0.12 × 0.10 mm
*V* = 1153.0 (5) Å^3^	

### Data collection

**Table:**

Bruker SMART APEX CCD diffractometer	3998 independent reflections
Radiation source: sealed tube	3255 reflections with *I* > 2σ(*I*)
Monochromator: graphite	*R*_int_ = 0.018
*T* = 293(2) K	θ_max_ = 25.1º
φ and ω scans	θ_min_ = 2.3º
Absorption correction: multi-scan(SADABS; Bruker, 2000)	*h* = −9→9
*T*_min_ = 0.985, *T*_max_ = 0.992	*k* = −10→10
5765 measured reflections	*l* = −19→16

### Refinement

**Table:**

Refinement on *F*^2^	Secondary atom site location: difference Fourier map
Least-squares matrix: full	Hydrogen site location: inferred from neighbouring sites
*R*[*F*^2^ > 2σ(*F*^2^)] = 0.046	H-atom parameters constrained
*wR*(*F*^2^) = 0.144	*w* = 1/[σ^2^(*F*_o_^2^) + (0.0904*P*)^2^ + 0.1284*P*] where *P* = (*F*_o_^2^ + 2*F*_c_^2^)/3
*S* = 1.00	(Δ/σ)_max_ < 0.001
3998 reflections	Δρ_max_ = 0.15 e Å^−^^3^
309 parameters	Δρ_min_ = −0.20 e Å^−^^3^
Primary atom site location: structure-invariant direct methods	Extinction correction: none

### Special details

**Table:**

Geometry. All e.s.d.'s (except the e.s.d. in the dihedral angle between two l.s. planes) are estimated using the full covariance matrix. The cell e.s.d.'s are taken into account individually in the estimation of e.s.d.'s in distances, angles and torsion angles; correlations between e.s.d.'s in cell parameters are only used when they are defined by crystal symmetry. An approximate (isotropic) treatment of cell e.s.d.'s is used for estimating e.s.d.'s involving l.s. planes.
Refinement. Refinement of *F*^2^ against ALL reflections. The weighted *R*-factor *wR* and goodness of fit *S* are based on *F*^2^, conventional *R*-factors *R* are based on *F*, with *F* set to zero for negative *F*^2^. The threshold expression of *F*^2^ > σ(*F*^2^) is used only for calculating *R*-factors(gt) *etc*. and is not relevant to the choice of reflections for refinement. *R*-factors based on *F*^2^ are statistically about twice as large as those based on *F*, and *R*-factors based on ALL data will be even larger.

### Fractional atomic coordinates and isotropic or equivalent isotropic displacement parameters (Å^2^)

**Table:**

	*x*	*y*	*z*	*U*_iso_*/*U*_eq_	
C1	0.3034 (2)	−0.11674 (18)	0.07248 (10)	0.0532 (4)	
C2	0.1837 (3)	−0.0094 (3)	0.06055 (17)	0.0920 (8)	
H2	0.0998	−0.0228	0.0332	0.110*	
C3	0.1875 (3)	0.1190 (3)	0.08930 (16)	0.0863 (7)	
H3	0.1066	0.1934	0.0805	0.104*	
C4	0.30938 (19)	0.13943 (17)	0.13103 (9)	0.0464 (4)	
C5	0.4274 (2)	0.02782 (18)	0.14337 (11)	0.0557 (4)	
H5	0.5097	0.0392	0.1721	0.067*	
C6	0.4257 (2)	−0.10127 (18)	0.11367 (11)	0.0579 (4)	
H6	0.5067	−0.1761	0.1217	0.069*	
C7	0.3015 (2)	0.28325 (18)	0.16136 (11)	0.0513 (4)	
H7A	0.3025	0.3681	0.1131	0.062*	
H7B	0.1946	0.2855	0.2031	0.062*	
C8	0.5758 (2)	0.39025 (17)	0.15325 (10)	0.0506 (4)	
H8	0.5780	0.4366	0.0964	0.061*	
C9	0.7086 (2)	0.41397 (18)	0.18741 (10)	0.0535 (4)	
C10	0.6996 (2)	0.34162 (18)	0.27232 (11)	0.0545 (4)	
H10	0.7867	0.3563	0.2973	0.065*	
C11	0.5642 (2)	0.24809 (17)	0.32079 (10)	0.0502 (4)	
C12	0.4356 (2)	0.23031 (17)	0.28237 (9)	0.0481 (4)	
H12	0.3430	0.1686	0.3134	0.058*	
C13	0.8551 (3)	0.5148 (3)	0.13244 (14)	0.0805 (6)	
H13A	0.8947	0.4865	0.0787	0.121*	
H13B	0.9460	0.5045	0.1613	0.121*	
H13C	0.8178	0.6177	0.1223	0.121*	
C14	0.5536 (3)	0.1662 (2)	0.41234 (11)	0.0665 (5)	
H14A	0.6636	0.1660	0.4245	0.100*	
H14B	0.5168	0.0643	0.4210	0.100*	
H14C	0.4735	0.2161	0.4499	0.100*	
C15	0.8272 (2)	0.8781 (2)	0.25321 (12)	0.0631 (5)	
C16	0.9551 (2)	1.0193 (2)	0.32804 (11)	0.0566 (4)	
C17	0.8784 (2)	0.88381 (18)	0.32838 (10)	0.0539 (4)	
C18	0.8522 (2)	0.76197 (17)	0.40146 (10)	0.0497 (4)	
C19	0.9004 (2)	0.77045 (17)	0.47720 (10)	0.0506 (4)	
H19	0.9526	0.8578	0.4785	0.061*	
C20	0.8724 (2)	0.65455 (17)	0.54774 (10)	0.0502 (4)	
H20	0.9058	0.6646	0.5961	0.060*	
C21	0.7936 (2)	0.51845 (17)	0.54952 (10)	0.0502 (4)	
C22	0.7464 (2)	0.50974 (18)	0.47347 (11)	0.0588 (4)	
H22	0.6953	0.4221	0.4718	0.071*	
C23	0.7742 (2)	0.62636 (19)	0.40311 (11)	0.0581 (4)	
H23	0.7409	0.6166	0.3547	0.070*	
C24	0.7631 (2)	0.39860 (18)	0.62288 (10)	0.0553 (4)	
C25	0.7996 (2)	0.41186 (19)	0.70048 (11)	0.0583 (4)	
C26	0.6899 (3)	0.2598 (2)	0.62445 (11)	0.0639 (5)	
N1	0.3014 (2)	−0.25176 (18)	0.03913 (9)	0.0650 (4)	
N2	0.44324 (16)	0.30155 (13)	0.20044 (8)	0.0456 (3)	
N3	0.7848 (3)	0.8728 (2)	0.19242 (12)	0.0903 (6)	
N4	1.0142 (2)	1.12948 (19)	0.33055 (11)	0.0752 (5)	
N5	0.8270 (3)	0.4254 (2)	0.76315 (10)	0.0802 (5)	
N6	0.6314 (3)	0.1475 (2)	0.62508 (12)	0.0907 (6)	
O1	0.1827 (2)	−0.2699 (2)	0.00906 (11)	0.0984 (5)	
O2	0.4200 (2)	−0.33952 (15)	0.04148 (8)	0.0797 (4)	

### Atomic displacement parameters (Å^2^)

**Table:**

	*U*^11^	*U*^22^	*U*^33^	*U*^12^	*U*^13^	*U*^23^
C1	0.0619 (10)	0.0509 (9)	0.0478 (9)	−0.0093 (7)	−0.0113 (7)	−0.0133 (7)
C2	0.0824 (14)	0.1014 (16)	0.134 (2)	0.0240 (12)	−0.0685 (14)	−0.0698 (15)
C3	0.0792 (14)	0.0869 (14)	0.1301 (19)	0.0341 (11)	−0.0692 (14)	−0.0587 (14)
C4	0.0492 (8)	0.0473 (8)	0.0439 (8)	−0.0011 (6)	−0.0155 (6)	−0.0082 (6)
C5	0.0644 (10)	0.0535 (9)	0.0587 (10)	0.0069 (7)	−0.0338 (8)	−0.0135 (7)
C6	0.0739 (11)	0.0473 (9)	0.0563 (10)	0.0070 (8)	−0.0274 (8)	−0.0089 (7)
C7	0.0539 (9)	0.0485 (8)	0.0569 (9)	0.0028 (7)	−0.0238 (7)	−0.0125 (7)
C8	0.0615 (10)	0.0445 (8)	0.0447 (8)	−0.0022 (7)	−0.0143 (7)	−0.0058 (6)
C9	0.0573 (10)	0.0478 (8)	0.0572 (9)	−0.0040 (7)	−0.0146 (8)	−0.0135 (7)
C10	0.0587 (10)	0.0546 (9)	0.0595 (10)	0.0006 (7)	−0.0241 (8)	−0.0207 (7)
C11	0.0617 (10)	0.0477 (8)	0.0453 (8)	0.0049 (7)	−0.0169 (7)	−0.0156 (7)
C12	0.0552 (9)	0.0449 (8)	0.0443 (8)	−0.0021 (6)	−0.0115 (7)	−0.0107 (6)
C13	0.0729 (13)	0.0809 (14)	0.0830 (14)	−0.0252 (11)	−0.0143 (11)	−0.0090 (11)
C14	0.0828 (13)	0.0734 (12)	0.0456 (9)	−0.0002 (9)	−0.0220 (9)	−0.0113 (8)
C15	0.0654 (11)	0.0626 (11)	0.0613 (11)	0.0035 (8)	−0.0258 (9)	−0.0042 (8)
C16	0.0548 (10)	0.0537 (10)	0.0570 (10)	0.0021 (8)	−0.0132 (8)	−0.0051 (7)
C17	0.0556 (9)	0.0501 (9)	0.0559 (9)	0.0043 (7)	−0.0179 (7)	−0.0083 (7)
C18	0.0522 (9)	0.0460 (8)	0.0529 (9)	0.0062 (7)	−0.0153 (7)	−0.0140 (7)
C19	0.0545 (9)	0.0458 (8)	0.0553 (9)	0.0005 (7)	−0.0146 (7)	−0.0176 (7)
C20	0.0570 (9)	0.0498 (8)	0.0484 (9)	0.0045 (7)	−0.0149 (7)	−0.0186 (7)
C21	0.0578 (9)	0.0460 (8)	0.0496 (9)	0.0056 (7)	−0.0141 (7)	−0.0161 (7)
C22	0.0759 (11)	0.0463 (9)	0.0603 (10)	−0.0065 (8)	−0.0261 (8)	−0.0129 (7)
C23	0.0762 (11)	0.0518 (9)	0.0544 (9)	0.0000 (8)	−0.0294 (8)	−0.0137 (7)
C24	0.0677 (10)	0.0481 (9)	0.0509 (9)	0.0010 (7)	−0.0141 (8)	−0.0130 (7)
C25	0.0734 (11)	0.0492 (9)	0.0490 (10)	0.0027 (8)	−0.0104 (8)	−0.0097 (7)
C26	0.0795 (12)	0.0543 (10)	0.0566 (10)	−0.0027 (9)	−0.0187 (9)	−0.0071 (8)
N1	0.0819 (11)	0.0605 (9)	0.0519 (8)	−0.0165 (8)	−0.0075 (7)	−0.0163 (7)
N2	0.0523 (7)	0.0422 (6)	0.0459 (7)	0.0010 (5)	−0.0171 (6)	−0.0121 (5)
N3	0.0981 (14)	0.1037 (14)	0.0760 (12)	0.0055 (11)	−0.0442 (11)	−0.0116 (10)
N4	0.0802 (11)	0.0611 (10)	0.0811 (11)	−0.0123 (8)	−0.0199 (9)	−0.0071 (8)
N5	0.1159 (15)	0.0736 (11)	0.0536 (9)	0.0017 (10)	−0.0239 (9)	−0.0160 (8)
N6	0.1217 (16)	0.0631 (11)	0.0885 (13)	−0.0249 (10)	−0.0333 (11)	−0.0059 (9)
O1	0.0935 (11)	0.1097 (12)	0.1151 (13)	−0.0195 (9)	−0.0270 (9)	−0.0635 (10)
O2	0.1179 (12)	0.0539 (7)	0.0688 (8)	0.0086 (8)	−0.0227 (8)	−0.0187 (6)

### Geometric parameters (Å, °)

**Table:**

C1—C2	1.356 (3)	C13—H13B	0.960
C1—C6	1.362 (2)	C13—H13C	0.960
C1—N1	1.469 (2)	C14—H14A	0.960
C2—C3	1.372 (3)	C14—H14B	0.960
C2—H2	0.930	C14—H14C	0.960
C3—C4	1.377 (2)	C15—N3	1.151 (2)
C3—H3	0.930	C15—C17	1.416 (2)
C4—C5	1.373 (2)	C16—N4	1.151 (2)
C4—C7	1.508 (2)	C16—C17	1.413 (2)
C5—C6	1.384 (2)	C17—C18	1.416 (2)
C5—H5	0.930	C18—C23	1.412 (2)
C6—H6	0.930	C18—C19	1.419 (2)
C7—N2	1.4818 (19)	C19—C20	1.357 (2)
C7—H7A	0.970	C19—H19	0.930
C7—H7B	0.970	C20—C21	1.419 (2)
C8—N2	1.343 (2)	C20—H20	0.930
C8—C9	1.379 (2)	C21—C24	1.408 (2)
C8—H8	0.930	C21—C22	1.421 (2)
C9—C10	1.388 (2)	C22—C23	1.359 (2)
C9—C13	1.509 (2)	C22—H22	0.930
C10—C11	1.386 (2)	C23—H23	0.930
C10—H10	0.930	C24—C25	1.418 (2)
C11—C12	1.379 (2)	C24—C26	1.420 (3)
C11—C14	1.505 (2)	C25—N5	1.147 (2)
C12—N2	1.3442 (19)	C26—N6	1.150 (2)
C12—H12	0.930	N1—O1	1.220 (2)
C13—H13A	0.960	N1—O2	1.220 (2)
			
C2—C1—C6	121.58 (16)	H13A—C13—H13C	109.5
C2—C1—N1	118.90 (16)	H13B—C13—H13C	109.5
C6—C1—N1	119.51 (16)	C11—C14—H14A	109.5
C1—C2—C3	119.27 (16)	C11—C14—H14B	109.5
C1—C2—H2	120.4	H14A—C14—H14B	109.5
C3—C2—H2	120.4	C11—C14—H14C	109.5
C2—C3—C4	121.06 (17)	H14A—C14—H14C	109.5
C2—C3—H3	119.5	H14B—C14—H14C	109.5
C4—C3—H3	119.5	N3—C15—C17	179.6 (2)
C5—C4—C3	118.37 (15)	N4—C16—C17	177.63 (19)
C5—C4—C7	124.51 (13)	C16—C17—C15	116.70 (15)
C3—C4—C7	117.11 (14)	C16—C17—C18	120.87 (14)
C4—C5—C6	120.97 (14)	C15—C17—C18	122.42 (15)
C4—C5—H5	119.5	C23—C18—C17	121.56 (14)
C6—C5—H5	119.5	C23—C18—C19	116.56 (14)
C1—C6—C5	118.74 (15)	C17—C18—C19	121.88 (14)
C1—C6—H6	120.6	C20—C19—C18	121.80 (14)
C5—C6—H6	120.6	C20—C19—H19	119.1
N2—C7—C4	113.85 (12)	C18—C19—H19	119.1
N2—C7—H7A	108.8	C19—C20—C21	121.66 (14)
C4—C7—H7A	108.8	C19—C20—H20	119.2
N2—C7—H7B	108.8	C21—C20—H20	119.2
C4—C7—H7B	108.8	C24—C21—C20	121.76 (14)
H7A—C7—H7B	107.7	C24—C21—C22	121.76 (14)
N2—C8—C9	121.24 (14)	C20—C21—C22	116.48 (14)
N2—C8—H8	119.4	C23—C22—C21	121.60 (15)
C9—C8—H8	119.4	C23—C22—H22	119.2
C8—C9—C10	117.15 (15)	C21—C22—H22	119.2
C8—C9—C13	119.71 (16)	C22—C23—C18	121.90 (15)
C10—C9—C13	123.14 (16)	C22—C23—H23	119.1
C11—C10—C9	121.71 (14)	C18—C23—H23	119.1
C11—C10—H10	119.1	C21—C24—C25	121.48 (14)
C9—C10—H10	119.1	C21—C24—C26	122.28 (14)
C12—C11—C10	117.85 (14)	C25—C24—C26	116.22 (15)
C12—C11—C14	119.55 (15)	N5—C25—C24	178.50 (19)
C10—C11—C14	122.60 (15)	N6—C26—C24	179.5 (2)
N2—C12—C11	120.51 (14)	O1—N1—O2	122.89 (16)
N2—C12—H12	119.7	O1—N1—C1	118.57 (17)
C11—C12—H12	119.7	O2—N1—C1	118.52 (15)
C9—C13—H13A	109.5	C8—N2—C12	121.53 (13)
C9—C13—H13B	109.5	C8—N2—C7	119.09 (12)
H13A—C13—H13B	109.5	C12—N2—C7	119.38 (13)
C9—C13—H13C	109.5		
			
C6—C1—C2—C3	1.1 (4)	C23—C18—C19—C20	−0.3 (2)
N1—C1—C2—C3	−178.1 (2)	C17—C18—C19—C20	178.78 (15)
C1—C2—C3—C4	−0.9 (4)	C18—C19—C20—C21	0.1 (2)
C2—C3—C4—C5	−0.1 (3)	C19—C20—C21—C24	−179.44 (15)
C2—C3—C4—C7	−179.1 (2)	C19—C20—C21—C22	0.3 (2)
C3—C4—C5—C6	0.9 (3)	C24—C21—C22—C23	179.20 (16)
C7—C4—C5—C6	179.83 (16)	C20—C21—C22—C23	−0.6 (3)
C2—C1—C6—C5	−0.4 (3)	C21—C22—C23—C18	0.4 (3)
N1—C1—C6—C5	178.90 (15)	C17—C18—C23—C22	−179.01 (16)
C4—C5—C6—C1	−0.7 (3)	C19—C18—C23—C22	0.0 (3)
C5—C4—C7—N2	4.9 (2)	C20—C21—C24—C25	4.2 (3)
C3—C4—C7—N2	−176.16 (17)	C22—C21—C24—C25	−175.56 (16)
N2—C8—C9—C10	0.3 (2)	C20—C21—C24—C26	−177.65 (16)
N2—C8—C9—C13	−179.80 (16)	C22—C21—C24—C26	2.6 (3)
C8—C9—C10—C11	0.7 (2)	C2—C1—N1—O1	−6.9 (3)
C13—C9—C10—C11	−179.16 (17)	C6—C1—N1—O1	173.77 (16)
C9—C10—C11—C12	−1.0 (2)	C2—C1—N1—O2	171.89 (19)
C9—C10—C11—C14	178.76 (15)	C6—C1—N1—O2	−7.4 (2)
C10—C11—C12—N2	0.2 (2)	C9—C8—N2—C12	−1.1 (2)
C14—C11—C12—N2	−179.49 (14)	C9—C8—N2—C7	178.29 (13)
C16—C17—C18—C23	179.39 (16)	C11—C12—N2—C8	0.8 (2)
C15—C17—C18—C23	0.5 (3)	C11—C12—N2—C7	−178.60 (13)
C16—C17—C18—C19	0.4 (2)	C4—C7—N2—C8	99.78 (16)
C15—C17—C18—C19	−178.44 (16)	C4—C7—N2—C12	−80.84 (18)

### Hydrogen-bond geometry (Å, °)

**Table:**

*D*—H···*A*	*D*—H	H···*A*	*D*···*A*	*D*—H···*A*
C5—H5···N2	0.93	2.56	2.895 (2)	102
C7—H7B···N4^i^	0.97	2.43	3.245 (3)	141
C8—H8···O2^ii^	0.93	2.46	3.119 (2)	128

Symmetry codes:  (i) *x*−1, *y*−1, *z*; (ii) *x*, *y*+1, *z*.

## References

[bb1] Bruker (2000). *SADABS*, *SMART* and *SAINT* Bruker AXS Inc., Madison, Wisconsin, USA.

[bb2] Liu, G. X., Ren, X. M., Kremer, P. K. & Meng, Q. J. (2005). *J. Mol. Struct.* **743**, 125–133.

[bb3] Madalan, A. M., Roesky, H. W., Andruh, M., Noltemeyer, M. & Stanica, N. (2002). *Chem. Commun.* pp. 1638–1639. 10.1039/b202628g 12170820

[bb4] Ren, X. M., Chen, Y. C., He, C. & Gao, S. (2002). *J. Chem. Soc. Dalton Trans.* pp. 3915–3918.

[bb5] Ren, X. M., Ma, J., Lu, C. S., Yang, S. Z., Meng, Q. J. & Wu, P. H. (2003). *Dalton Trans.* pp. 1345–1351.

[bb6] Ren, X. M., Meng, Q. J., Song, Y., Lu, C. S., Hu, C. J. & Chen, X. Y. (2002). *Inorg. Chem.* **41**, 5686–5692. 10.1021/ic0111637 12401072

[bb7] Sheldrick, G. M. (2008). *Acta Cryst.* A**64**, 112–122. 10.1107/S0108767307043930 18156677

[bb8] Wang, P.-F., Liu, G.-X. & Chen, Y.-C. (2006). *Acta Cryst.* E**62**, o3256–o3258.

